# The First Two Cases of *Candida* *auris* in The Netherlands

**DOI:** 10.3390/jof5040091

**Published:** 2019-09-30

**Authors:** Erik H. Vogelzang, Annemarie J.L. Weersink, Rosa van Mansfeld, Nancy A. Chow, Jacques F. Meis, Karin van Dijk

**Affiliations:** 1 Department of Medical Microbiology and Infection Control, Amsterdam UMC, location VUmc, 1081 HV Amsterdam, The Netherlands; r.vanmansfeld@amsterdamumc.nl (R.v.M.); k.vandijk1@amsterdamumc.nl (K.v.D.); 2 Department of Medical Microbiology and Immunology, Meander Medical Center, 3813 TZ Amersfoort, The Netherlands; AJL.Weersink@meandermc.nl; 3 Mycotic Diseases Branch, Division of Foodborne, Waterborne and Environmental Diseases, National Center for Emerging and Zoonotic Infectious Diseases, Centers for Disease Control and Prevention, Atlanta, GA 30333, USA; yln3@cdc.gov; 4 Department of Medical Microbiology and Infectious Diseases, Canisius Wilhelmina Hospital (CWZ), 6532 SZ Nijmegen, The Netherlands; jacques.meis@gmail.com; 5 Centre of Expertise in Mycology, Radboudumc/CWZ, 6532 SZ Nijmegen, The Netherlands

**Keywords:** *Candida auris*, emergence, molecular typing, infection prevention

## Abstract

*Candida auris* is a rapidly emerging multidrug-resistant pathogenic yeast. In recent years, an increasing number of *C. auris* invasive infections and colonized patients have been reported, and *C. auris* has been associated with hospital outbreaks worldwide, mainly in intensive care units (ICUs). Here, we describe the first two cases of *C. auris* in The Netherlands. Both cases were treated in a healthcare facility in India prior to admission. The patients were routinely placed in contact precautions in a single room after admission, which is common practice in The Netherlands for patients with hospitalization outside The Netherlands. No transmission of *C. auris* was noticed in both hospitals. Routine admission screening both for multidrug-resistant (MDR) bacteria and MDR yeasts should be considered for patients admitted from foreign hospitals or countries with reported *C. auris* transmission.

## 1. Introduction

Since the first report of *Candida auris* in 2009, an increasing number of *C. auris* invasive infections and colonized patients have been reported in at least 37 countries and territories, including the USA, Africa, Europe, and Asia [1,2,3,4,5,6,7,8]. *C. auris* has been associated with outbreaks in hospitals and other healthcare facilities. Ongoing transmission despite enhanced infection prevention and control measures during nosocomial outbreaks has been reported in several studies [4,5]. Resistance of *C. auris* to several antifungal classes of drugs with few having high minimum inhibitory concentrations (MICs) to all major drug classes has been described [9,10,11]. The nosocomial outbreaks and multidrug resistance of *C. auris* are worrisome and call for enhanced hospital infection prevention measures [12,13]. Previous *C. auris* outbreak investigations showed that co-colonization with multidrug-resistant (MDR) bacteria is common in critically ill patients [14]. In The Netherlands, admission screening, consisting of cultures from the nose, throat, and rectum, of all patients who were admitted to a foreign hospital for more than 24 hours is still only focused on MDR gram-negative bacteria and methicillin-resistant *Staphylococcus aureus* (MRSA). Concern is raised regarding the surveillance for MDR organisms after admission to foreign hospitals, since screening for MDR yeasts is not routinely included in The Netherlands. We report the first two cases of *C. auris* in The Netherlands, in patients who had healthcare exposures in hospitals in India. 

## 2. Materials and Methods

Case 1 was a middle-aged man repatriated from a hospital in India to an intensive care unit (ICU) in The Netherlands. He had been hospitalized for five weeks in India for treatment of sepsis with multiple organ failure due to pneumonia. He was mechanically ventilated with a tracheostomy tube and continuous renal replacement therapy for which he had a central venous catheter. Since he was repatriated from a foreign healthcare facility, contact precautions and nursing in a private room were imposed according to standard practice in The Netherlands. Routine admission screening cultures for methicillin-resistant *Staphylococcus aureus* (MRSA) and MDR gram-negative bacteria taken from nose, throat, and rectum did not show growth of yeasts but demonstrated colonization with MDR *Enterobacterales*, producing OXA 48 and NDM. Although there were no signs of infection during admission in The Netherlands, the central venous catheter (CVC) was cultured after removal. Sheep blood agar (bioMérieux, Marcy l’Etoile, France) showed growth of yeast colonies that were identified with Matrix Assisted Laser Desorption Ionization-Time of Flight mass spectrometry (MALDI-TOF; Bruker, Bremen, Germany, database version MBT Compass 4.1) as *C. auris*. Since there were no signs and symptoms of infection, antifungal therapy was not initiated. Further screening during admission (axilla, groin, rectum, and oropharynx) of the patient for *C. auris* on CHROMOagar (BD) revealed the presence of *C. auris* in the groin. Cultures after discharge were negative for *C. auris*. No specific screening of the *C. auris* patient contacts or surveillance was performed since on hospital arrival the patient was placed in contact precautions in a single-patient room. But *C. auris* has not been cultured in clinical samples or routine selective digestive decontamination (SDD) screening samples since then.

Case 2 was a middle-aged man referred by the Amsterdam airport medical services due to shortness of breath and an inability to stand without assistance. He was in the Amsterdam airport for a layover while travelling back to the USA from India. Among others, he had a history of diabetes mellitus type 2 and renal insufficiency for which he received hemodialysis. Up to two days prior to admission, he had received hemodialysis in a healthcare facility in India. Therefore, contact precautions in a private room were implemented and screening cultures for MRSA and MDR gram-negative bacteria were obtained. Cultures demonstrated colonization with several MDR gram-negative bacteria such as OXA and NDM-positive *Escherichia coli* and NDM positive *Pseudomonas aeruginosa*. Therefore, more stringent cleaning and disinfection in the single-patient room was implemented, which consisted of terminal cleaning and disinfection with hydrogen peroxide: incidin oxy (Ecolab, Monheim am Rhein, Germany) instead of cleaning alone.

Due to a low-grade fever, one day after admission, a urine sample was obtained. In the urine culture, 10^4^–10^5^ colony-forming units of smooth beige yeasts grew on Sabouraud dextrose agar supplemented with 50mg/L chloramphenicol (produced in-house). The colonies were identified as *C. auris* using MALDI-TOF (bioMérieux, database version 3.2). Antifungal therapy was not initiated since the patient did not have clinical signs of a urinary tract infection. On the ward where the patient had been admitted, we performed a surveillance screening for *C. auris* of patients using CHROMagar Candida (Becton Dickinson, Franklin Lakes, NJ, USA), including axilla, groin, rectum, oropharynx, and if applicable, catheter urine and wounds; no *C. auris* was detected during screening.

## 3. Results

### Laboratory Investigations

*C. auris* in vitro susceptibility was performed according to the Clinical and Laboratory Standards Institute reference method for broth dilution of yeast [15]. Broth microdilution showed that both *C. auris* isolates had high fluconazole (>64 mg/L) and voriconazole (4 mg/L) minimum inhibitory concentrations (MICs). Amphotericin B (0.5–1 mg/L), anidulafungin (<0.016–0.063 mg/L), and micafungin (0.063 mg/L) had low MICs. Molecular typing with amplified fragment length polymorphism and microsatellite analysis showed that both isolates belonged to the South Asian *C. auris* Clade I (not shown). 

**Figure 1:**
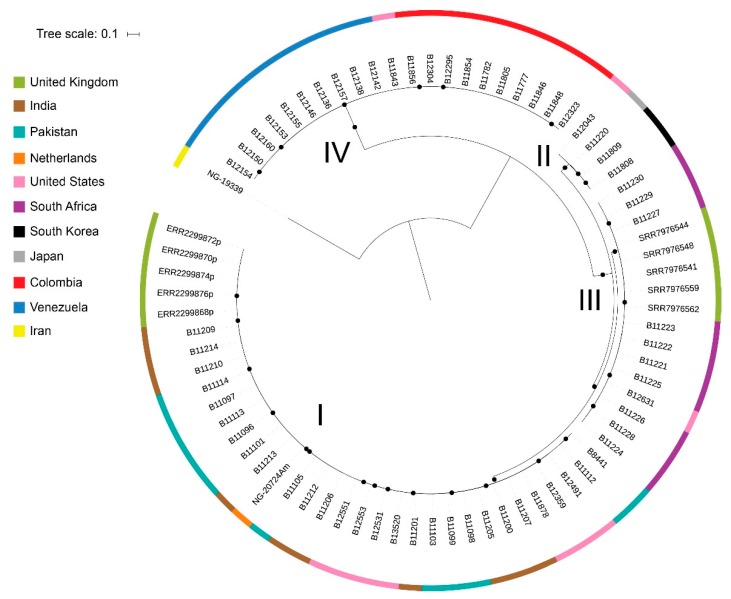
Description of *C. auris* major clades. Maximum likelihood phylogenetic tree of isolates from *C. auris* cases from 11 countries. Circles at nodes indicate separations with a bootstrap value ≥99%. I: South Asian (Clade I), II: East Asian (Clade II), III: African Clade (Clade III), and IV: South American (Clade IV).

**Figure 2:**
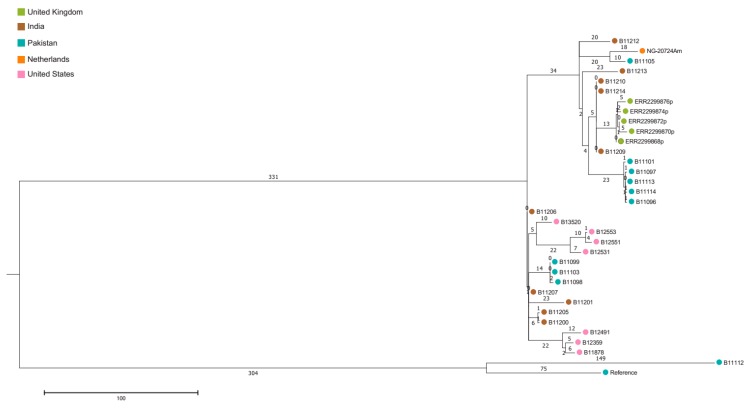
Description of South Asian Clade I. Maximum parsimony phylogenetic tree of isolates from The Netherlands, India, Pakistan, UK, and the USA.

Since it was not clear whether case 2 had already colonized with *C. auris* contracted during his multiple stays in long-term care facilities in the New York area or had contracted the colonization during his hemodialysis in India, we compared whole genome sequencing (WGS) of this isolate (NG-20724Am) with published WGS sequences from the USA. Genomic DNA was extracted from 2-day-old colonies as described earlier [13]. Genomic libraries for WGS were constructed and sequenced using Illumina technology (Illumina, San Diego, CA, USA) at Eurofins Genomics (Ebersberg, Germany). Seventy-four *C. auris* WGS sequences retrieved from NCBI were added to the analysis. FastQC and PRINSEQ were used to assess the quality of read data and perform read filtering. Read data were aligned against a publicly available genome sequenced on PacBio RS II using BWA [7]. SNP variants were identified using SAMtools and filtered using the publicly available SNP analysis pipeline NASP to remove positions that had less than 10x coverage, less than 90% variant allele calls, or that were identified by Nucmer as being within duplicated regions in the reference. Phylogenetic analysis and bootstrapping with 500 iterations was performed on SNP matrices. The raw sequence read files were uploaded to the NCBI Sequence Read Archive and are publicly available under BioProject ID: PRJNA560710 (Submission ID: SUB6189371). Figure 1 and Figure 2 show the isolate from case 2 clustering with isolates from India and those from the USA, which have been argued to be a result of *C. auris* introductions from South Asia followed by local transmission [7].

## 4. Discussion

Here we present the first two imported cases of *C. auris* in The Netherlands and the first whole-genome sequences of a *C. auris* isolate from The Netherlands. Both patients were placed in contact precautions and admission screening samples were taken from the nose, oropharynx, and rectum since both patients had recently been admitted to a healthcare facility in India. During initial admission screening in The Netherlands, no specific effort was taken to detect *C. auris*. However, clinical samples taken during hospitalization showed growth of *C. auris* from a central venous line tip and from urine cultures. No additional cases of *C. auris* in clinical samples have been identified at both medical centers in over a year since the detection of *C. auris* in case 1 and 2. Since not all cultured yeasts are identified, there is a possibility that transmission has occurred. However, we believe that since a large proportion of cultures with yeast are identified to species level, a nosocomial outbreak would not remain unnoticed. Screening of axilla and groins after the clinical samples became positive identified skin colonization in case 1, but fortunately the implementation of admission contact precautions for MDR may have prevented hospital transmission of *C. auris* in both cases. Similar scenarios were recently reported from the USA [14,16]. This suggests that routine screening for *C. auris* should be strongly considered in the general admission workup when a patient is admitted to a hospital in The Netherlands from a foreign healthcare facility, especially from endemic regions such as South Asia, the Middle East, and South Africa but also transfers from facilities in Europe with *C. auris* endemic transmission as seen in some hospitals in Spain and the UK. 

Both *C. auris* isolates exhibited high azole MICs, which is in accordance with the reported literature [3,17,18,19]. Reduced susceptibility to amphotericin B has also been demonstrated [18,19,20], and therefore, echinocandins are the recommended empirical treatment for *C. auris* [21,22]. However, although rare, reduced susceptibility of echinocandins to *C. auris* has been reported [6]. Fortunately, in the absence of an infection no antifungal treatment was initiated in our cases. 

Due to its antifungal resistance, association with nosocomial outbreaks, and for the potential prevention of outbreaks when identified early, there is a rationale for screening for *C. auris*. However, there is limited evidence and experience with screening for *C. auris*. The Centers for Disease Control and Prevention (CDC) states that screening could be considered for close healthcare contacts with newly identified *C. auris* infection or colonization and patients who have had an overnight stay in a healthcare facility outside the USA in the previous year, especially in countries with documented *C. *auris** cases [23]. The European Centre for Disease Prevention and Control (ECDC) has similar recommendations [24]. However, little is known of the prevalence of *C. auris* patient colonization in various countries and hospital settings (e.g., ICU, ICU with a nosocomial outbreak, or long-term care facilities). Most studies reporting colonization are during nosocomial outbreaks. During a nosocomial outbreak in an ICU in the UK, 63 out of 900 (7%) screened patients were colonized, and 7 patients were infected (0.7%) [5]. In addition, New York State health officials are considering mandatory pre-admission screening for *C. auris* since in various healthcare facilities in New York there is an ongoing *C. auris* outbreak [2]. A study investigating this outbreak reported *C. auris* colonization in 61 of 581 patients screened (10.4%) [2]. In a recent study in ICUs in India, *C. auris* represented 5.2% of all candidemia cases [25], and *C. auris* has become the second most isolated pathogen at a large trauma center [26]. Therefore, *C. auris* colonization of patients in ICUs in India is expected to be substantially higher. Based on the above described prevalence, there is a rationale for routine admission screening for *C. auris* in patients from healthcare facilities (especially ICUs) in India, from healthcare facilities in which nosocomial transmission has been reported, and other high prevalence areas (although there is limited information about what the other high prevalence areas are). 

When screening for *C. auris*, it is not yet defined which body sites should be included. The CDC advises screening patients’ bilateral axilla and groin. In addition to the axilla and groin, the ECDC suggests that other sites (urine, wounds, catheter exit sites, throat, and other locations) can be sampled if indicated. During a large *C. auris* outbreak in an ICU, screening of patients involved the nose, axilla, groin, urine culture, and if present, tracheostomy and wounds [5]. In patients colonized with *C. auris*, the first positive screening result was from the axilla in 22 of 60 patients (37%), from another site (groin or urine) in 21 of 60 (35%), and from both the axilla and one or more other sites in 17 of 60 (28%). No other studies describe the colonization sites of *C. auris* specifically. The high percentage of patients first colonized in the axilla is probably due to the fact that the hospital outbreak was found to be linked to reusable axillary temperature probes, and therefore, the generalizability of this result is probably limited [5]. 

Only one study investigated the sensitivity of a single screen for *C. auris* [5]. Patients underwent screening twice within two days: 62 of 79 screening samples (78%) after a first positive screen were positive again. The authors concluded that a single screen was not sensitive enough to detect colonization with *C. auris*. More studies are needed before a recommendation about the number of screenings necessary to adequately detect *C. auris* can be made. In endemic regions, the implementation of a rapid and automated molecular surveillance admission screening for *C. auris* may be considered [27,28]. The advantage in endemic regions would be that these sensitive tests allow rapid diagnosis and may therefore prevent spread. In non-endemic areas, most tests will be negative and thus automated molecular surveillance admission screening will probably be not cost-effective.

*C. auris* has been isolated in numerous countries on five continents, and WGS have revealed 5 distinct clades that represent the following geographical regions: South Asia (India/Pakistan), South Africa, South America, East Asia, and the Middle East [6,29]. Both our cases recently returned from India, and molecular typing showed that the isolates belonged to the South Asian *C. auris* Clade I. Two recent studies from the USA demonstrated that the majority of detected *C. auris* clustered with the South Asian clade [2,7]. Interestingly, our second case was living in the USA and therefore could have been colonized with *C. auris* clones from healthcare facilities in the USA (mainly in the New York area). By using a maximum parsimony phylogenetic tree of isolates, WGS has the potential to identify the origin of imported cases and could thereby identify populations relevant for targeted screening. With WGS, this isolate clustered with isolates from both the Indian subcontinent and isolates from the USA. 

In summary, since *C. auris* is potentially multidrug resistant, is associated with nosocomial outbreaks, and outbreaks can potentially be prevented when identified early by implementing contact precautions, the performance of routine admission screening both for MDR bacteria and yeasts should be considered for patients admitted from foreign hospitals or countries with reported *C. auris* transmission.
